# The Ribosome Restrains Molten Globule Formation in Stalled Nascent Flavodoxin*

**DOI:** 10.1074/jbc.M116.756205

**Published:** 2016-10-26

**Authors:** Joseline A. Houwman, Estelle André, Adrie H. Westphal, Willem J. H. van Berkel, Carlo P. M. van Mierlo

**Affiliations:** From the Laboratory of Biochemistry, Wageningen University, 6708 WE Wageningen, The Netherlands

**Keywords:** flavin mononucleotide (FMN), protein folding, protein misfolding, ribosome, translation, molten globule, ribosome-nascent chain complex

## Abstract

Folding of proteins usually involves intermediates, of which an important type is the molten globule (MG). MGs are ensembles of interconverting conformers that contain (non-)native secondary structure and lack the tightly packed tertiary structure of natively folded globular proteins. Whereas MGs of various purified proteins have been probed to date, no data are available on their presence and/or effect during protein synthesis. To study whether MGs arise during translation, we use ribosome-nascent chain (RNC) complexes of the electron transfer protein flavodoxin. Full-length isolated flavodoxin, which contains a non-covalently bound flavin mononucleotide (FMN) as cofactor, acquires its native α/β parallel topology via a folding mechanism that contains an off-pathway intermediate with molten globular characteristics. Extensive population of this MG state occurs at physiological ionic strength for apoflavodoxin variant F44Y, in which a phenylalanine at position 44 is changed to a tyrosine. Here, we show for the first time that ascertaining the binding rate of FMN as a function of ionic strength can be used as a tool to determine the presence of the off-pathway MG on the ribosome. Application of this methodology to F44Y apoflavodoxin RNCs shows that at physiological ionic strength the ribosome influences formation of the off-pathway MG and forces the nascent chain toward the native state.

## Introduction

Proteins need to fold into their correct native conformations to perform their functions. Folding progresses from unfolded to natively folded protein and often involves intermediates (1, 2). Some of these intermediates are dead ends within the folding funnel (*i.e.* local minima in free energy) and must therefore (partially) unfold before the productive folding pathway can be followed (3). Molten globules are a specific type of folding intermediates, which contain secondary structure elements but lack the tight tertiary packing found in natively folded proteins. Molten globular intermediates can reside on or off the folding pathway to native protein. MGs^2^ are prone to aggregation and consequently are implicated in various diseases (4).

Since the first description of MGs (5), much research has focused on determining conditions at which MGs form. Several proteins form these intermediates under mildly alkaline or acidic conditions (6–9). Currently, many proteins are thought to fold via MG intermediates (10–13). For example, it has been postulated that the occurrence of MG species is necessary during insertion of proteins into membranes or through membrane pores (14–16). Such insertions often happen co-translationally, *i.e.* while the ribosome synthesizes the protein concerned. It has been shown that proteins can fold co-translationally and sample intermediate folding states (17–23), which might include MGs.

Determining the ability of the proteins to form co-translational MGs would be a stepping-stone in elucidating protein folding in the cell, and potentially, it contributes to understanding the initiation of protein aggregation. Characterization of MGs is complicated by their often transient nature, generally low presence at equilibrium, conformational heterogeneity, and the tendency to aggregate (24). The presence of MGs can be revealed through use of extrinsic dyes, such as thioflavin T (ThT), as these dyes become highly fluorescent upon binding to exposed hydrophobic residues (25). Other methods for MG detection include circular dichroism and intrinsic tryptophan fluorescence (26–28). However, these methodologies are unsuitable for studying co-translational MG formation, as ribosome-nascent chain complexes (RNCs) not only contain the emerging polypeptide but also more than 50 ribosomal proteins. Thus, detection of MG formation on the ribosome is notoriously difficult.

**FIGURE 1.**
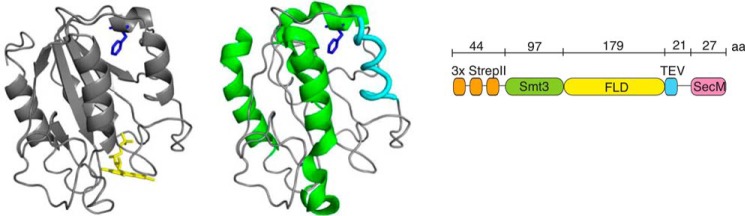
**Differences between flavodoxin's native and MG structure and the construct used for RNC production.** *Left*, schematic drawing of native flavodoxin (Protein Data Bank entry 1YOB (29)) with FMN colored *yellow*. Residue Phe-44 (*blue*) is shown in *stick* representation. *Middle*, schematic drawing of the four transiently structured regions of the off-pathway MG, which include α-helices (colored *green*) and structure that is neither α-helix nor β-strand (colored *cyan*) (33). These regions dock non-natively and form the core of apoflavodoxin's MG (33, 35, 39, 40). The schematic is a representation of structural elements in the MG and not of their packing. *Right*, construct for RNC production contains a triple N-terminal StrepII tag (*orange*), an Smt3 domain (*green*) fused to flavodoxin (*yellow*), a recognition site for TEV protease (*blue*), and a linker that spans the ribosomal exit tunnel and concludes with the SecM stalling sequence (*magenta*).

To investigate whether nascent chains form MGs, we utilize a 179-residue flavodoxin from *Azotobacter vinelandii*. Flavodoxin contains a non-covalently bound flavin mononucleotide (FMN) as cofactor and is involved in electron transport in the nitrate reduction cycle. The native protein consists of five α-helices sandwiching a central parallel β-sheet, which is an α/β parallel topology (Fig. 1, *left*) (29). According to Structural Classification of Proteins in the Protein Data Bank, approximately 25% of proteins share this topology. The flavodoxin-like fold is an ancestral fold (30) and is archetypal for the class of αβα sandwiches. It has been well established that proteins with a flavodoxin-like architecture fold *in vitro* through involvement of an off-pathway intermediate (11, 31–37). Upon folding, the large majority of unfolded flavodoxin molecules temporarily misfold. This misfolding yields an off-pathway MG that is prone to aggregation (38). In contrast to native protein, this MG contains no β-sheet and is helical (Fig. 1, *middle*) (33, 39, 40). Under steady-state conditions, the MG is in equilibrium with native apoflavodoxin. Before formation of native apoflavodoxin can occur, the MG has to (partially) unfold. This unfolding is the rate-limiting step in folding of native apoflavodoxin. The cofactor FMN only binds to the flavin-binding site of natively folded apoflavodoxin (41). This binding can be followed in time by fluorescence spectroscopy, as FMN's fluorescence becomes severely quenched upon binding to native apoflavodoxin (42, 43).

Upon mixing FMN with apoflavodoxin under conditions where MG is present, cofactor binding and accompanied fluorescence quenching should be delayed, as the MG needs to unfold before native apoflavodoxin can form. This delayed FMN quenching is used in this study to reveal whether the ribosome modulates formation of molten globular apoflavodoxin. Previously, we demonstrated that once apoflavodoxin has been entirely synthesized and is exposed outside the ribosome, the protein is natively folded and capable of binding FMN (44). To demonstrate whether MGs form during translation, we take advantage of the following folding characteristic of flavodoxin variant F44Y (39); the apo-form of this variant switches from natively folded to the off-pathway MG upon decreasing ionic strength to physiological values (39, 40). Because of the higher thermodynamic stability of the holo-form of this protein, once apoflavodoxin binds FMN this topological switching no longer occurs (45). We prepared stalled RNC complexes that expose the entire F44Y flavodoxin protein outside the ribosomal exit tunnel. The translational stalling of the ribosomes is caused by an *Escherichia coli* SecM-derived peptide (46, 47), which we attached at the C terminus of flavodoxin. After purification of F44Y apoflavodoxin RNCs, we determined the corresponding FMN binding rates and thus the influence of the ribosome on MG formation.

## Results

### 

#### 

##### Dependence of Apoflavodoxin's Off-pathway MG on Ionic Strength and Temperature

**FIGURE 2.**
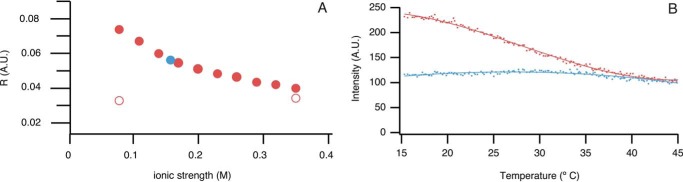
**Effects of ionic strength or temperature on F44Y apoflavodoxin.** *A*, ionic strength dependence of fluorescence anisotropy of F44Y (*closed red circles*) and C69A apoflavodoxin (*open red circles*) (graph adapted from Ref. 39). Upon decreasing ionic strength F44Y apoflavodoxin forms a MG, which is characterized by fluorescence anisotropy that is higher than native apoflavodoxin. Expected anisotropy of F44Y apoflavodoxin in buffer I is shown as a *blue circle. B*, temperature dependence of ThT binding to F44Y apoflavodoxin, as followed by fluorescence emission at 485 nm. Protein is in 10 (*red*) or 100 mm (*blue*) potassium PP_i_, pH 6.0. ThT binding to the MG of F44Y apoflavodoxin increases ThT fluorescence. Because of unfolding of the MG with increasing temperature, ThT releases, and its fluorescence decreases. Protein concentration is 3.3 μm, and the heating rate is 1 °C/min.

Decreasing salt concentration is a well known method to destabilize folded proteins (48). Using this approach in F44Y apoflavodoxin samples, we can shift the equilibrium between natively folded protein and MG to the latter species (39). Fluorescence anisotropy shows that lowering ionic strength from 100 to 10 mm potassium PP_i_ (*i.e.* 345 to 75 mm equivalent NaCl units) at 25 °C forces F44Y apoprotein from native apoflavodoxin to the MG (Fig. 2*A*). The MG state is characterized by fluorescence anisotropy that is higher than native apoflavodoxin. In contrast, at high salt concentrations, as for example 100 mm potassium PP_i_, F44Y apoflavodoxin is predominantly in its native state. For C69A apoflavodoxin, which is similar to wild-type apoflavodoxin (49, 50), no salt-dependent switch between folding states is observed (Fig. 2*A*), as its native state is considerably more stable than native F44Y apoflavodoxin (39).

The formation of the off-pathway MG not only depends on ionic strength but also on temperature. To follow the temperature-dependent existence of this MG, we added the extrinsic dye ThT. This reporter molecule is commonly employed to follow amyloid fibril formation, as it has a high affinity for β-sheets. Upon binding to fibrillar structures, ThT shows a fluorescence increase at 480 nm upon excitation at 450 nm. Binding of ThT and the subsequent fluorescence increase is not only restricted to fibrillar structures but can also occur due to the presence of non-β-sheet cavities, such as may form in MGs (25). Another extrinsic dye commonly used to probe MGs is 1-anilino-8-naphthalene sulfonate; however, in the case of F44Y apoflavodoxin, its fluorescence does not follow the unfolding of the MG, whereas ThT fluorescence does (data not shown).

Fig. 2*B* shows how temperature affects F44Y apoflavodoxin at high and low ionic strengths (*i.e.* 345 and 75 mm equivalent NaCl units, respectively). Temperature was raised from 15 to 45 °C, upon which F44Y apoflavodoxin unfolds (39). Temperature was not raised further to avoid formation of aggregates, which would lead to a rise in ThT fluorescence. For protein at low ionic strength and low temperature, ThT fluorescence is high (Fig. 2*B*), because F44Y apoflavodoxin is predominantly in the MG state. Upon increasing temperature, ThT fluorescence decreases non-cooperatively, because the MG unfolds gradually and binding of ThT diminishes. This gradual, non-cooperative unfolding is a typical feature of apoflavodoxin's MG (35, 37). Tryptophan fluorescence shows that the thermal unfolding transition of native F44Y apoflavodoxin in 100 mm potassium PP_i_ ranges from about 20 to 40 °C, with a thermal midpoint of 32.9 ± 0.3 °C (39). Above ∼40 °C, ThT fluorescence of protein at low and high salt concentrations is similar, because at both conditions the protein is now unfolded. At a high salt concentration, a slight hump in ThT fluorescence is observed in the thermal unfolding transition of native protein (Fig. 2*B*). This hump arises because the MG state of F44Y apoflavodoxin populates. At the thermal midpoint of native apoflavodoxin (*i.e.* 32.9 ± 0.3 °C), ThT fluorescence due to MG population is low because the MG is gradually unfolded.

Fig. 2*B* implies that at a low salt concentration, the MG of F44Y apoflavodoxin is maximally populated at about 15 °C. This corroborates our previous findings that the off-pathway MG of F44Y apoflavodoxin is structured in the absence of denaturant (35, 40).

##### FMN Binding Rate as a Tool to Reveal the Presence of Apoflavodoxin's MG

FMN is essential for flavodoxin's role in electron transport. This cofactor binds to native apoflavodoxin through a very specific combination and geometry of aromatic and hydrogen bond interactions (41, 45). In native apoflavodoxin, the flavin-binding site is flexible, whereas upon cofactor incorporation it becomes rather rigid (45, 51, 52). Cofactor binding not only influences the binding pocket itself but also affects residues far away, resulting in picomolar binding affinity of FMN (45). As a consequence, flavodoxin is thermodynamically very stable and has to first release FMN before it can unfold (41, 45). Accordingly, FMN binding to native apoflavodoxin is the last step during folding of flavodoxin. FMN neither acts as a nucleation site for folding nor does it interact with folding intermediates of apoflavodoxin, including the discussed off-pathway MG (41).

**FIGURE 3.**
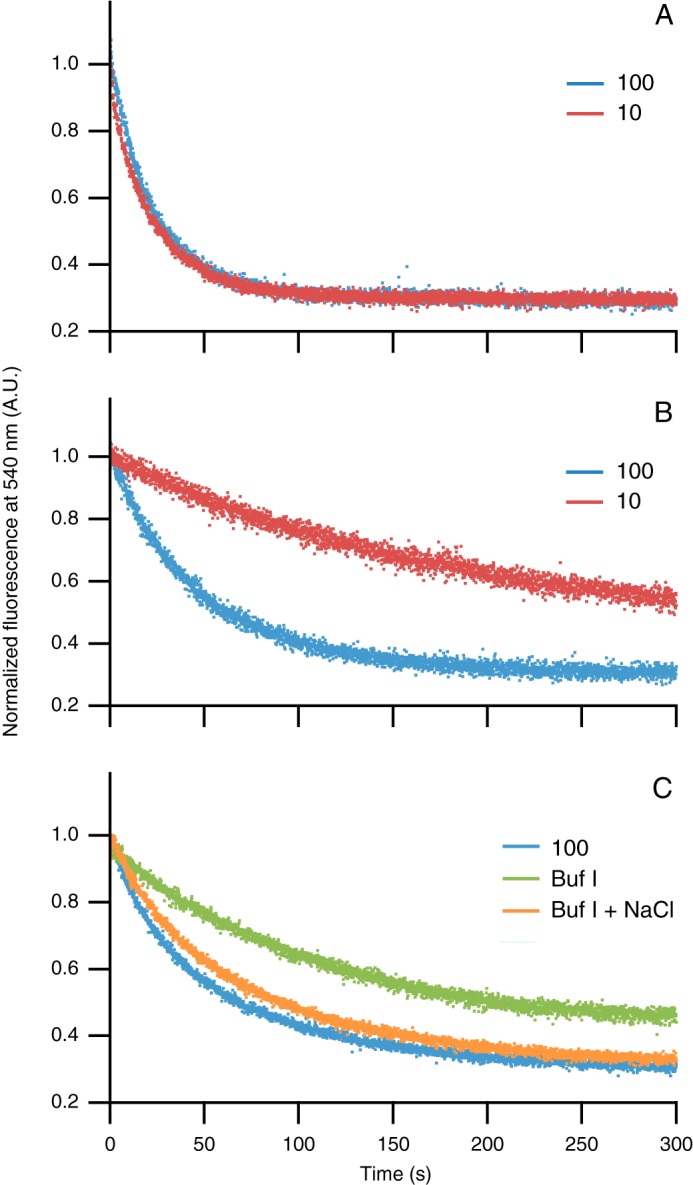
**Rate with which FMN binds to apoflavodoxin depends on the presence of molten globular protein and thus on the salt concentration.** To follow quenching of flavin fluorescence, FMN is added to protein at 25 °C. Shown are normalized FMN fluorescence traces. *A*, C69A apoflavodoxin in 100 (*blue*) and 10 (*red*) mm potassium PP_i_, pH 6.0. C69A protein is natively folded under both conditions and thus binds FMN with the same rate. Concentrations for protein and FMN are 140 and 25 nm, respectively. *B*, F44Y apoflavodoxin in 100 (*blue*) and 10 (*red*) mm potassium PP_i_, pH 6.0. In 100 mm potassium PP_i_, F44Y protein is natively folded, whereas in 10 mm potassium PP_i_ the F44Y variant predominantly populates the MG state, which delays FMN binding as the MG needs to unfold before native apoflavodoxin can form. Concentrations for F44Y apoflavodoxin and FMN are 71 and 25 nm, respectively. *C*, F44Y apoflavodoxin in 100 mm potassium PP_i_ (*blue*) and in buffer I (*Buf I*) containing 290 mm NaCl (*orange*). Protein is natively folded under these conditions and thus binds FMN with similar rate. F44Y apoflavodoxin in buffer I (*green*) populates the MG state, thereby delaying FMN binding. Concentrations of F44Y apoflavodoxin and FMN are 62 and 25 nm respectively.

Binding of FMN to native apoflavodoxin can be followed by FMN fluorescence, as FMN incorporation into native apoflavodoxin severely quenches its fluorescence. We demonstrate this phenomenon in Fig. 3*A*, which shows the time-dependent decrease of FMN fluorescence upon cofactor binding to C69A apoflavodoxin in 100 and 10 mm potassium PP_i_ at 25 °C. The rates of FMN binding at both salt concentrations are identical, because C69A apoflavodoxin is natively folded under these circumstances (39).

In contrast to C69A apoflavodoxin, the rates of FMN binding to F44Y apoflavodoxin in 100 and 10 mm potassium PP_i_ differ drastically (Fig. 3*B*). This dissimilarity arises because F44Y apoflavodoxin in 100 mm potassium PP_i_ is natively folded, whereas in 10 mm potassium PP_i_ the protein extensively populates the off-pathway MG. As this MG needs to unfold before productive folding can take place, FMN binding is delayed significantly when compared with F44Y apoflavodoxin in 100 mm potassium PP_i_ and C69A apoflavodoxin in 10 or 100 mm potassium PP_i_, respectively. Thus, ascertaining the rate of FMN binding to apoflavodoxin as a function of ionic strength is a suitable tool to detect the presence of apoflavodoxin's off-pathway MG.

To preserve ribosomal integrity during purification of RNCs, we use buffer I (50 mm HEPES-KOH, 100 mm potassium acetate, 15 mm magnesium acetate, 1 mm dithiothreitol (DTT), pH 7.4), which contains magnesium to hold the two ribosomal subunits together. At the ionic strength of buffer I, which is similar to that of the cellular environment, the MG state of F44Y apoflavodoxin populates (Fig. 2*A*). We therefore assessed the rate of FMN binding to F44Y apoflavodoxin in buffer I (Fig. 3*C*). When compared with F44Y apoflavodoxin in 100 mm potassium PP_i_, the FMN binding rate is considerably reduced in buffer I, indicating the presence of significant amounts of MG. To confirm that ionic strength also affects the equilibrium between MG and native apoflavodoxin in buffer I, 290 mm NaCl was added to buffer I, which would approximate the ionic strength of 100 mm potassium PP_i_. Indeed, the FMN binding rate increases upon addition of salt to buffer I and becomes comparable with the rate found for native apoflavodoxin.

##### Production and Purification of RNCs of F44Y Flavodoxin in Vivo

To assess whether MGs form during translation, we produced RNCs of C69A and F44Y flavodoxin in *E. coli* (called C69A_RNC_ and F44Y_RNC_, respectively), using the construct of Fig. 1, *right*. Production at 37 °C and purification of C69A_RNC_ have been described elsewhere (44). In case of F44Y_RNC_, we use the same procedure, except that now RNCs are induced in *E. coli* at either 37 or 15 °C. Lowering the temperature to 15 °C maximizes population of the MG state of F44Y apoflavodoxin, whereas at 37 °C the protein is unfolded (Fig. 2*B*).

**FIGURE 4.**
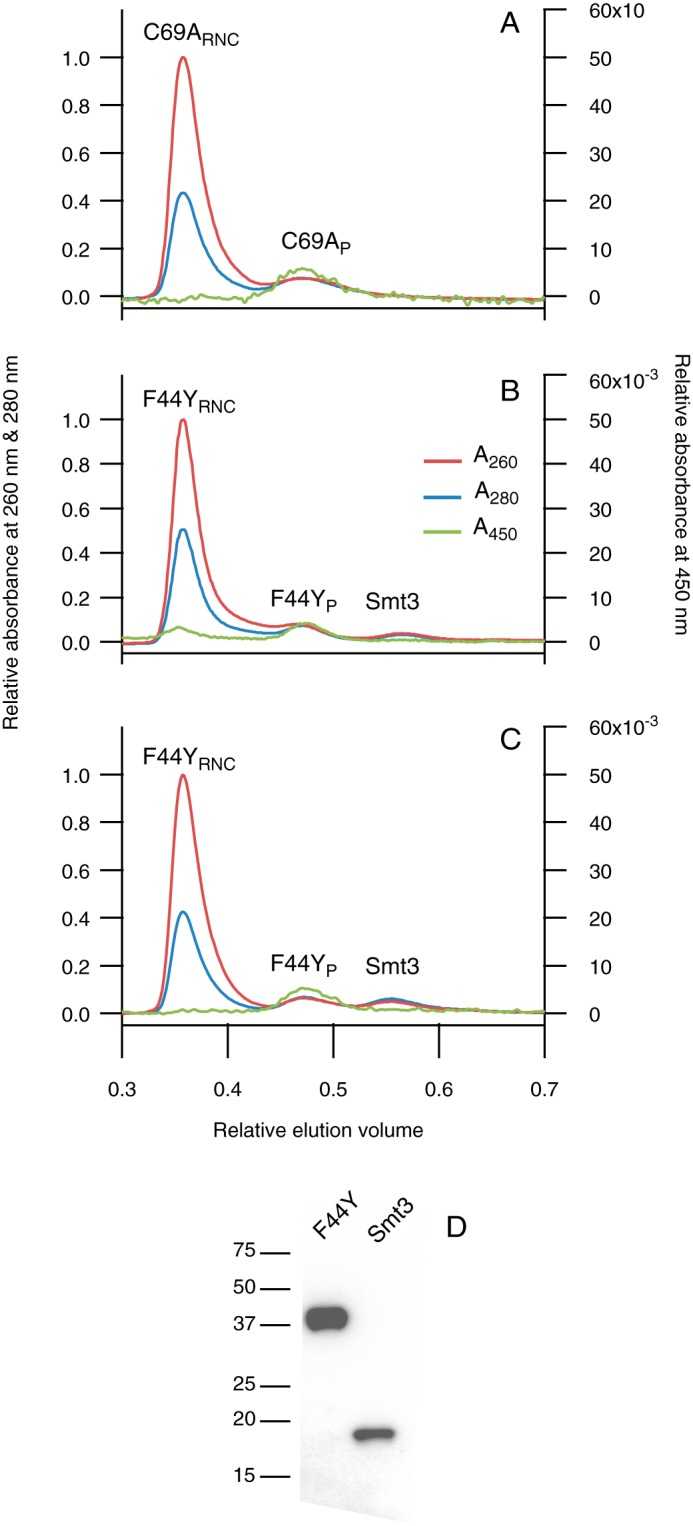
**Comparison of release and degradation of C69A and F44Y flavodoxin nascent chains produced in *E. coli* under several growth conditions**. Shown are Superdex75 elution profiles, with absorbance at 260 (*red*), 280 (*blue*), and 450 nm (*green*). All absorbance traces are normalized relative to the maximum absorbance at 260 nm of the respective RNCs. Absorbance at 450 nm tracks FMN incorporation. Elution volumes are normalized to the total column volume *V_t_*. All constructs are induced for 2.5 h in *E. coli* Δ*tig*::*kan* in minimal medium at either 37 °C (*A* and *B*) or 15 °C (*C*). *A*, use of C69A flavodoxin construct produces C69A_RNC_ and C69A_P_. *B*, use of F44Y flavodoxin construct produces F44Y_RNC_ and F44Y_P_. Proteolytic degradation of F44Y protein construct results in the presence of the fragment triple StrepII tag-Smt3 (labeled *Smt3*). *C*, use of F44Y flavodoxin construct at 15 °C produces F44Y_RNC_ and F44Y_P_ with the same degradation pattern (*i.e.* Smt3) as observed in (*B*). *D*, Western blot of the fractions labeled F44Y_P_ and Smt3 probed with antibodies against the N-terminal StrepII tag.

Although the SecM sequence allows for relatively tight stalling (46), some release of nascent chains happens during their production in *E. coli*, as we observed previously for RNCs of C69A flavodoxin (44). This release is most probably due to the physical force that is exerted as soon as the nascent chain folds (53, 54). Binding of FMN to natively folded nascent chains does not dissociate nascent flavodoxin from the ribosome (44). To separate released protein (called C69A_P_ or F44Y_P_) from RNCs (*i.e.* C69A_RNC_ or F44Y_RNC_), we utilize size-exclusion chromatography. The absorbance ratio *A*_260_:*A*_280_ enables distinction between RNCs and released flavodoxin, as this ratio is ∼2 for RNCs, although *A*_260_:*A*_280_ is <1 for released protein. The RNCs elute in the void volume of the Superdex75 column, whereas released protein elutes later. Fig. 4*A* illustrates this separation of C69A_RNC_ and C69A_P_ through size exclusion. A similar distribution is observed for the F44Y flavodoxin variant grown at 37 °C (Fig. 4*B*), showing that also in case of this protein variant release of nascent chains happens. However, an additional third elution peak is now present. This extra peak arises from the protein fragment consisting of the triple StrepII tag and Smt3 domain (Fig. 4, *B* and *C*), which we refer to as the Smt3 fragment. The identity of the 18-kDa fragment is confirmed by Western blotting probed with antibodies against the StrepII tag (Fig. 4*D*). Because the remaining C-terminal fragments no longer contain a StrepII tag, they are not present after the purification step using a Strep-Tactin column. We previously observed the same degradation product for C-terminally shortened nascent chains of C69A flavodoxin. These shortened proteins are less stable relative to the full-length C69A protein (44), and thus Fig. 4, *B* and *C,* also indicates decreased stability of both F44Y_RNC_ and/or F44Y_P_ when produced at 15 or 37 °C. Whether intracellular degradation of the F44Y construct occurs while it is bound to the ribosome and/or while it has been released is unknown. We can only distinguish between both species after their separation using size-exclusion chromatography. Since degradation had already happened, we were unable to determine which species degraded and formed the observed Smt3 fragment.

Production of the F44Y flavodoxin construct at 15 °C does not alter the elution peak arising from the Smt3 fragment (Fig. 4*C*). Thus, a temperature change of 37 to 15 °C has no effect on the amount of *in vivo* proteolytic degradation of F44Y_RNC_ and/or F44Y_P_, suggesting that the respective proteolytic susceptibility of nascent chain and released protein is similar at both temperatures. The ratio between F44Y_P_ and F44Y_RNC_ is calculated taking into account the differences in extinction coefficients at 280 nm of RNCs and released protein. At both temperatures the ratio is around 70, which is comparable with the ratio we have found previously for a C-terminally shortened nascent chain of C69A flavodoxin (44).

##### All Purified F44Y_P_ Contains FMN

**FIGURE 5.**
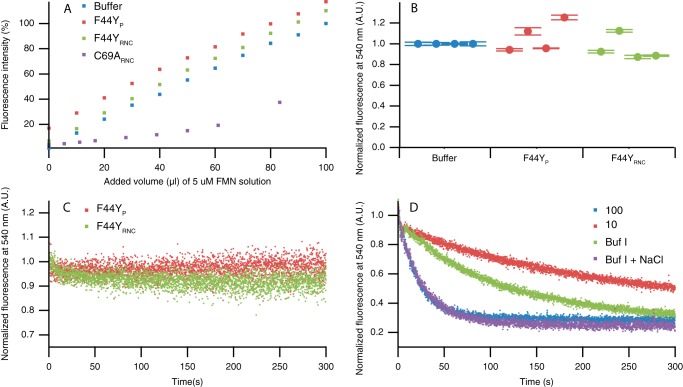
**RNCs of F44Y flavodoxin construct and released protein construct isolated from *E. coli* are saturated with FMN.** *A*, titration of buffer I, F44Y_P_, F44Y_RNC_, and C69A_RNC_ with FMN. Observation of an altering slope in FMN fluorescence titration data reveals cofactor binding, as is the case for C69A_RNC_. F44Y_P_ and F44Y_RNC_ show no change in slope of FMN fluorescence titration data. All fluorescence is normalized to the fluorescence of the end point of the titrated buffer sample. Concentration of protein and RNC is 0.5 μm. Data of C69A_RNC_ are adapted from Ref. 44. *B*, fluorescence of FMN in supernatants of F44Y_P_ (*red*) and F44Y_RNC_ (*green*) after TCA precipitation. The average fluorescence of TCA samples of four purifications is shown, together with the corresponding standard deviation. Before addition of TCA, concentration of F44Y_P_ and F44Y_RNC_ was 50 nm. Fluorescence is normalized to fluorescence of buffer I containing 50 nm FMN (*blue*). *C*, FMN binding traces of F44Y_P_ and F44Y_RNC_ isolated from *E. coli*. FMN fluorescence is normalized. F44Y_P_, F44Y_RNC_, and FMN concentrations are 50, 50, and 25 nm, respectively. *D*, triple Strep tag, Smt3 domain, the linker, and the SecM sequence of the construct used for RNC production do not influence the formation of the off-pathway MG of apoflavodoxin. Shown are FMN binding traces of apo-F44Y_P_ in 100 mm potassium PP_i_ (*blue*), 10 mm potassium PP_i_ (*red*), buffer I (*green*), and buffer I (*Buf I*) + 290 mm NaCl (*purple*). F44Y_P_ and FMN concentrations are 147 and 25 nm, respectively.

In a previous study, we showed that when FMN binds to a released C-terminally shortened C69A flavodoxin construct, this construct is protected against intracellular proteolysis, due to the increased thermodynamic stability conferred by incorporated FMN (44). As a consequence, all of these released constructs were saturated with FMN, because the unstable apo-forms are proteolytically degraded in *E. coli* (causing the appearance of the Smt3 fragment). In contrast, for C69A_P_, which is full-length protein construct, the FMN content varies between 40 and 100%, depending on the *E. coli* batch used for purification (44). Because of its increased thermodynamic stability compared with C-terminally shortened constructs, the apo-form of C69A_P_ is not proteolytically degraded. To verify in this study whether the elution peak labeled F44Y_P_ arises from protein saturated with cofactor, we titrated this fraction with FMN (Fig. 5*A*). Because of quenching of FMN fluorescence upon cofactor binding to apoprotein, the slope of fluorescence data arising from FMN bound to protein is less steep than the slope resulting from free FMN. This characteristic is widely used to demonstrate FMN binding (42, 43, 45). Fig. 5*A* reveals similar slopes for FMN fluorescence titration data of buffer and F44Y_P_. This observation shows that F44Y_P_ binds no additional FMN.

To determine the ratio of FMN to apoprotein, we added trichloroacetic acid (TCA) to F44Y_P_. This dissociates the cofactor from the protein, and after centrifugation, we measured FMN fluorescence of the supernatant. This experiment demonstrates equimolar presence of FMN and apoprotein (Fig. 5*B*). Full saturation of F44Y_P_ with FMN was seen independent of production temperature (*i.e.* 15 or 37 °C). Finally, we followed FMN fluorescence in time upon addition of the cofactor to F44Y_P_ (Fig. 5*C*). If the sample would contain any F44Y_P_ in its apo-form, one should detect a decrease in FMN fluorescence upon FMN binding. However, no such decrease was observed and thus no cofactor binding happened.

In conclusion, binding of FMN protects destabilized apoflavodoxin variants against the action of intracellular proteases, and thus all F44Y_P_ we purify contains FMN.

##### F44Y_RNC_ Isolated from E. coli Is Saturated with FMN

To determine the extent of cofactor incorporation into F44Y_RNC_, we performed a titration of the purified nascent protein with FMN. Fig. 5*A* shows that no added FMN binds to these RNCs. TCA precipitation of RNCs shows that this observation arises because RNCs of F44Y flavodoxin contain FMN (Fig. 5*B*), regardless of whether they are produced by *E. coli* at 15 or 37 °C. We observe only a marginal decrease in FMN fluorescence after addition of the cofactor to F44Y_RNC_ (Fig. 5*C*). This decrease corresponds to maximally 5% of F44Y_RNC_ being in the apo-form. In contrast to the observed nearly full saturation of F44Y_RNC_ with FMN, we previously demonstrated that C69A_RNC_ is purified from *E. coli* as apoprotein. Observation of an altering slope in FMN fluorescence titration data shows that C69A_RNC_ binds FMN (Fig. 5*A*). This difference in FMN occupation of F44Y_RNC_ and C69A_RNC_, and the observation that F44Y_P_ is fully saturated with FMN, suggests that when the apo-form of F44Y protein is present in *E. coli*, it is not as stable in the cellular environment of *E. coli* as C69A apoprotein is. Therefore, only the holo-forms of RNCs and released F44Y protein are purified.

##### Domains of F44Y_P_ Do Not Affect MG Formation of Apoflavodoxin

To assess whether the triple Strep tag, the Smt3 domain, the linker, and the SecM sequence (Fig. 1, *right*) influence MG formation of F44Y apoflavodoxin, we removed FMN from purified F44Y_P_ according to a previously published procedure (44). Subsequently, the FMN binding trace of this refolded apo-F44Y_P_ was measured in various buffers (Fig. 5*D*). Comparison of the refolded apo-F44Y_P_ traces with those of F44Y apoflavodoxin (Fig. 3*C*) shows that for both proteins a change in ionic strength has a similar effect on FMN binding. Increasing potassium PP_i_ concentration from 10 to 100 mm, or addition of 290 mm NaCl to buffer I, leads to an increase in FMN binding rate. This observation is due to increased population of the native state of apoflavodoxin. Upon addition of salt (buffer I with 290 mm NaCl), the FMN binding rate of apo-F44Y_P_ is similar to the rate observed for apo-F44Y_P_ in 100 mm potassium PP_i_, as both proteins are natively folded. Thus, the presence of the triple Strep tag, the Smt3 domain, the linker, and the SecM sequence of the construct used for RNC production does not influence formation of the off-pathway MG of apoflavodoxin or impair FMN binding.

##### Ribosome Forces Entirely Synthesized and Fully Exposed F44Y Apoflavodoxin toward the Native State

Because no apo-F44Y_RNC_ could be obtained from *E. coli* due to proteolytic degradation, we adopted an *in vitro* transcription/translation approach to generate nascent F44Y constructs in their apo-form. We used a highly pure *in vitro* protein synthesis kit (PURExpress® Δ (aa, tRNA), New England Biolabs) to preclude the presence of FMN in the translation reaction. We avoided usage of *E. coli* extracts for *in vitro* protein synthesis, as FMN is difficult to remove from these extracts. The highly purified *in vitro* protein synthesis kit does not contain chaperones that interact with nascent chains, such as trigger factor and DnaK.

**FIGURE 6.**
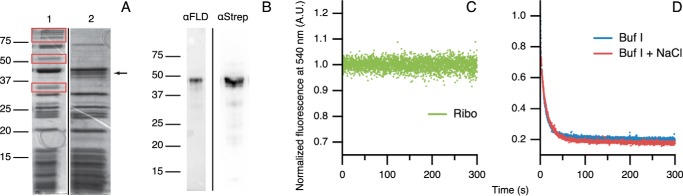
**Apoflavodoxin in F44Y_RNC_ is natively folded at low and high salt concentrations.** *A,* Coomassie-stained gels of samples taken from an *in vitro* translation reaction of F44Y flavodoxin construct before (*lane 1*) and after (*lane 2*) purification with Strep-Tactin affinity chromatography. An *arrow* labels the position of F44Y flavodoxin construct, and *red boxes* enclose the bands of the non-ribosomal components of the *in vitro* translation reaction. *B,* Western blotting of sample (2), probed with antibodies against flavodoxin (α*FLD*) or N-terminal StrepII tag (α*Strep*), respectively. *C,* FMN fluorescence shows that ribosomes (Ribo) do not bind FMN. Concentrations of ribosomes and FMN are 50 and 25 nm, respectively. *A.U.,* arbitrary units. *D,* FMN binding traces of F44Y_RNC_ in buffer I (*blue*) and in this buffer with 290 mm NaCl (*red*). Concentrations of F44Y_RNC_ and FMN are 50 and 25 nm, respectively.

*In vitro* protein synthesis yields a very low percentage of stalled ribosomes, as can be inferred from Fig. 6*A*. Shown is a Coomassie-stained gel of a sample from an *in vitro* translation reaction before and after purification with Strep-Tactin column chromatography. The gel of the sample before purification shows protein bands originating from ribosomal proteins. Also, bands arising from non-ribosomal components of the *in vitro* translation kit, like aminoacyl-tRNA synthases, can be seen. These non-ribosomal components can be removed by Strep-Tactin column chromatography. In Fig. 6*A* the position of the band corresponding to F44Y protein construct is indicated. The identity of this band is verified by Western blottings probed with antibodies against flavodoxin and StrepII tag (Fig. 6*B*). Before purification of the *in vitro* translation mixture, this band was barely visible, as most ribosomes were devoid of stalled nascent chains. After Strep-Tactin chromatography purification, the intensity of this band was comparable with those of ribosomal proteins, because in stalled RNCs all proteins are present in equimolar quantities.

In contrast, F44Y flavodoxin nascent chains produced in *E. coli* are released from the ribosome in considerable quantities, as described above (Fig. 4, *B* and *C*). Interestingly, the amount of F44Y_P_ produced *in vitro* is very small, because, as mentioned, Fig. 6*A* shows that the Coomassie-stained bands of F44Y flavodoxin RNCs are all of comparable intensities. In case of nascent chain release, the released construct would have been purified together with the remaining RNCs by Strep-Tactin chromatography. Essentially, F44Y flavodoxin construct would be overproduced and would therefore no longer be equimolar to ribosomal proteins, which is clearly not the case (Fig. 6*A*).

As discussed, determination of the FMN binding rate enables detection of the presence of apoflavodoxin's MG. This methodology is thus suitable to investigate formation of molten globular nascent apoflavodoxin, provided that ribosomes themselves do not bind FMN. To check the latter, FMN was added to purified *E. coli* ribosomes, and its fluorescence followed in time. As can be inferred from Fig. 6*C*, FMN does not associate with ribosomes, because no change in flavin fluorescence is observed.

We assessed the rate of FMN binding to F44Y_RNC_ in buffer I and in this buffer with 290 mm NaCl. Fig. 6*D* shows that this increase in salt concentration does not affect the rate of FMN binding to F44Y_RNC_. In contrast, such salt concentration change of buffer I considerably increases the rate of FMN binding to F44Y apoflavodoxin (Fig. 3*C*) and to the apo-form of F44Y_P_ (Fig. 5*D*), as F44Y apoflavodoxin switches from MG to native protein. Because F44Y_RNC_ binds FMN rapidly at both salt concentrations, apoflavodoxin in F44Y_RNC_ must be natively folded under both conditions. We note that another implication of this observation is that the sample of F44Y_RNC_ cannot contain the released F44Y flavodoxin construct, because if this would be the case the mentioned increase in salt concentration would lead to a change in FMN binding rate. Such a change is not seen in Fig. 6*D*, thereby corroborating the above-mentioned equimolar presence of nascent chain and ribosomal proteins. At low ionic strength, F44Y apoflavodoxin and the apo-form of F44Y_P_ are molten globular, but, remarkably, apoflavodoxin in F44Y_RNC_ is natively folded. Apparently, the ribosome modulates flavodoxin folding and forces F44Y apoflavodoxin that is entirely synthesized and exposed outside the ribosome, to which it is stalled by an artificial linker containing the SecM sequence, toward the native state. To our knowledge, this is the first time the effect of the ribosome on formation of MGs during protein synthesis has been determined.

## Discussion

Determination of the rate of flavin binding is an innovative approach that makes it possible to investigate the presence of MGs during translation of flavin-binding proteins. Using this methodology, we demonstrated that the ribosome forces nascent and fully exposed F44Y apoflavodoxin toward the native state. Confinement of MG formation during translation is an important observation that emphasizes differences between folding *in vivo* and *in vitro*.

Because of the presence of ribosomal RNA surrounding the end of the exit tunnel, this part of the ribosome area has a considerable negative surface charge (55). Although some proteins only seem to randomly interact with the ribosomal surface (56), others interact to such an extent that local motions of compact domains are constrained (57) or folding/unfolding transitions are slowed down (58). These interactions likely arise due to attraction of positively charged residues and simultaneous repulsion of negatively charged residues of the nascent chain by the negatively charged ribosomal outer surface. Because the population of natively folded apoflavodoxin within F44Y_RNC_ at a low salt concentration (*i.e.* in buffer I) is higher than is the case for F44Y apoflavodoxin in buffer I, the ribosome restrains formation of the off-pathway MG. Upon release of the nascent chain this effect is negated, as F44Y_P_, a construct that was stalled to the ribosome before its release due to physical exertion, can form the MG state upon lowering ionic strength (Fig. 5*D*). It is tempting to speculate that the ribosome potentially mimics the effects of high ionic strength, thereby forcing molten globular apoflavodoxin to the native state. Native apoflavodoxin has a net charge of −13 at neutral pH (59). Possibly, the conformational space of unfolded nascent apoflavodoxin is restricted due to electrostatic repulsion of the nascent chain by the ribosomal surface, leading to entropic stabilization of native protein at physiological ionic strength.

We note that the RNC construct we use creates a somewhat artificial situation compared with translation of solely the flavodoxin gene. Because of the presence of the SecM sequence and attached linker (Fig. 1, *right*), RNCs are produced with the entire flavodoxin domain exposed outside the ribosome. In contrast, when a ribosome reaches the stop codon and terminates the nascent chain during translation of solely the flavodoxin domain, ∼30–40 residues of the protein are still buried in the exit tunnel. These residues need to traverse this tunnel before full-length protein emerges from the ribosome. We showed based on investigating C-terminally shortened flavodoxin RNCs that exposure of the five C-terminal residues of flavodoxin is essential for the nascent chain to be able to attain its native fold (44). Thus, although the 30–40 C-terminal residues of flavodoxin are inside the exit tunnel, the part of the protein that is already outside the exit tunnel is unable to fold natively.

It would be interesting to verify whether our finding that the ribosome forces flavodoxin toward the native state also applies to the folding of flavodoxin-like domains within multidomain proteins like cytochrome P450 reductase and nitric-oxide synthase (60, 61). As each domain of such a protein is translated the nascent chain remains tethered to the ribosome, thus providing the ribosome ample opportunity to influence the folding of an already translated flavodoxin-like domain.

## Experimental Procedures

### 

#### 

##### Protein Expression and Purification

To avoid covalent dimerization of purified *A. vinelandii* (apo)flavodoxin, the single cysteine at position 69 was substituted by an alanine (50). The C69A variant is similar to wild-type (apo)flavodoxin (49, 50). An additional F44Y mutation was introduced (39), and this protein variant is referred to as F44Y (apo)flavodoxin. Both constructs were transformed in *E. coli* (strain TG2) and purified as described previously (50). Apoprotein was prepared according to established protocols (44). Apoprotein concentrations were determined by titration with known amounts of FMN. Purified proteins in 100 mm potassium pyrophosphate (potassium PP_i_) buffer, pH 6.0, were flash-frozen in liquid nitrogen and stored at −80 °C.

Because of cold denaturation, some F44Y apoflavodoxin deteriorates into soluble molecules that are incapable of binding FMN upon thawing. These molecules cannot be separated from FMN binding-competent apoflavodoxin, and thus the latter concentration was determined by titration with FMN in 100 mm potassium PP_i_, pH 6.0. Also, as much as possible, freshly prepared F44Y apoflavodoxin was used in experiments.

##### Influence of Temperature on Full-length Isolated Protein

Thermal unfolding of F44Y apoflavodoxin was probed by ThT fluorescence using a Cary Eclipse spectrophotometer (Varian). Excitation was at 445 nm, and emission was measured at 485 nm. Temperature was increased from 15 to 45 °C at a rate of 1 °C/min. The excitation and emission slits were set to bandwidths of 5 and 10 nm, respectively, and the PMT voltage was 900 V. 3.3 μm F44Y apoflavodoxin and 56 μm ThT (Sigma) were used in 10 or 100 mm potassium PP_i_, pH 6.0.

##### Engineering RNC Variants

Plasmids for RNC production contain a sequence coding for a triple N-terminal StrepII tag, an Smt3 domain, a TEV site, a linker, and a SecM stalling motif (Fig. 1, *right*). The flavodoxin sequence (C69A or F44Y) was inserted in the multiple cloning site present between the Smt3 domain and the TEV site. Ulp1 protease specifically recognizes the Smt3 domain and cleaves downstream from this domain, thereby producing nascent flavodoxin chains with Ala-1 as the authentic N terminus, if required. The recognition site for TEV protease enables release of nascent protein from the ribosome. The plasmid was a kind gift of Professor Elke Deuerling (University of Konstanz, Germany) (56).

##### Expression and Purification of RNCs in Vivo and in Vitro

Plasmids were transformed in *E. coli* strain BL21(DE3) Δ*tig*::*kan* for expression of RNCs *in vivo*. All RNC variants were prepared as described previously (44). After growth of *E. coli* in Terrific Broth medium, cells were transferred to minimal (M9) medium for expression of RNCs at either 37 or 15 °C for 1 h. RNCs were purified using Strep-Tactin-Sepharose (IBA GmbH) chromatography and subsequent size-exclusion (Superdex75) chromatography. RNCs and released protein chains were purified and stored in buffer I (50 mm HEPES-KOH, 100 mm potassium acetate, 15 mm magnesium acetate, 1 mm dithiothreitol (DTT), pH 7.4). After purification, the apo-form of released protein chains of F44Y flavodoxin was prepared in 100 mm potassium PP_i_, pH 6.0, as described previously (44).

For *in vitro* expression of RNCs, the PURExpress® Δ (aa, tRNA) *in vitro* protein synthesis kit from New England Biolabs was used. The manufacturer's instructions were followed. After mixing the kit's components with the F44Y flavodoxin containing RNC plasmid, the reaction was allowed to proceed for 2 h at 37 °C. To purify RNCs produced *in vitro*, the reaction mixture was loaded onto a Strep-Tactin-Sepharose column and eluted as described previously in buffer I (44). Eluted RNCs were concentrated with a 10-kDa spin filter (EMD Millipore) at 5500 × *g*. Concentrated samples were frozen in liquid nitrogen and stored at −80 °C. Samples of each purification step were analyzed by Coomassie-stained SDS-PAGE or Western blotting using either flavodoxin antibody raised in rabbits (Eurogentec) followed by anti-rabbit HRP-IG (Eurogentec) or StrepMAB classic (IBA GmbH) against the N-terminal StrepII tag.

##### FMN Fluorescence

To establish FMN content of RNCs and released constructs, FMN titration or TCA precipitation was performed as described previously (44). To determine the MG nature of RNCs of F44Y protein and of released protein, binding of FMN to these molecules was followed in time. All flavin fluorescence experiments were measured on a Cary Eclipse spectrophotometer (Varian) at 25 °C.

Samples for FMN titration contained 0.5 μm of either RNC or released protein produced *in vivo* in buffer I. Samples were titrated with an FMN solution of 5 μm to determine their cofactor-binding capacity using flavin fluorescence. Excitation was at 450 nm, with emission recorded between 500 and 600 nm. For each titration point, the average of five scans was taken. Excitation and emission slits were set to a bandwidth of 5 nm, and PMT voltage was set to 900 V.

Samples for TCA precipitation contained 50 nm RNC or released protein produced *in vivo* in buffer I. To establish the total amount of FMN bound to protein, the samples were precipitated by adding 3% (w/v) TCA. Precipitate was spun down for 10 min at 21,000 × *g* at 4 °C, and the supernatant was carefully pipetted off to measure its flavin fluorescence. A 50 nm solution of FMN in 3% (w/v) TCA in buffer I was used as reference. Excitation was at 450 nm, with emission recorded between 500 and 600 nm. Each sample was scanned five times, and the average was used for calculations. Excitation and emission slits were set to a bandwidth of 10 or 20 nm, respectively. PMT voltage was set to 970 V.

To determine the rate of FMN binding to C69A and F44Y variants of apoflavodoxin, *E. coli* ribosomes (New England Biolabs), RNC, or released protein, samples were made of each with protein concentrations ranging from 50 to 147 nm. Samples were measured in stirred fluorescence cuvettes for 5 min before and after adding FMN to a final concentration of 24 nm. Excitation was at 450 nm, and emission was monitored at 540 nm. Excitation and emission slits were set to bandwidths of 10 and 20 nm, respectively, and PMT voltage was 970 V. The averaging time of the measurement was 0.1 s. The time between addition of FMN and the start of fluorescence detection was ∼2 s. Buffers used in these experiments were 10 mm potassium PP_i_, pH 6.0, 100 mm potassium PP_i_, pH 6.0, buffer I or buffer I with a final concentration of 290 mm NaCl. The latter buffer has an ionic strength equal to that of 100 mm potassium PP_i_, pH 6.0.

## Author Contributions

J. A. H., E. A., and A. H. W. expressed and purified flavodoxin variants and ribosomal nascent chain complexes. J. A. H. and A. H. W. acquired and analyzed the data. J. A. H., W. J. H. v. B., and C. P. M. v. M. wrote the manuscript.

## Footnotes

2 The abbreviations used are:

MG: molten globule
RNC: ribosome-nascent chain
ThT: thioflavin T
TEV: tobacco etch virus.
